# Supplementary material for: Modeling glioblastoma heterogeneity as a dynamic network of cell states
Source: Mol Syst Biol. 2021 Sep 16;17(9):e10105. doi: 10.15252/msb.202010105 (PMC8444284; doi:10.15252/msb.202010105)
Supplement: Supplementary file 5 — Source Data for Figure 3 [file MSB-17-e10105-s001.zip › Figure3A_sourcedata/GSEA_3065/hallmarks_state5.GseaPreranked.1623416302988/gsea_report_for_na_pos_1623416302988.html]

Report for na\_pos 1623416302988 [GSEA]

| GS  follow link to MSigDB | GS DETAILS | SIZE | ES | NES | NOM p-val | FDR q-val | FWER p-val | RANK AT MAX | LEADING EDGE || 1 | HALLMARK\_ANGIOGENESIS | Details ... | 21 | 0.74 | 2.20 | 0.000 | 0.000 | 0.000 | 1304 | tags=62%, list=15%, signal=72% |
| 2 | HALLMARK\_EPITHELIAL\_MESENCHYMAL\_TRANSITION | Details ... | 119 | 0.52 | 2.16 | 0.000 | 0.000 | 0.000 | 1399 | tags=50%, list=16%, signal=58% |
| 3 | HALLMARK\_INFLAMMATORY\_RESPONSE | Details ... | 69 | 0.43 | 1.64 | 0.004 | 0.029 | 0.053 | 2175 | tags=48%, list=24%, signal=63% |
| 4 | HALLMARK\_CHOLESTEROL\_HOMEOSTASIS | Details ... | 60 | 0.40 | 1.49 | 0.018 | 0.072 | 0.161 | 1389 | tags=38%, list=15%, signal=45% |
| 5 | HALLMARK\_MYOGENESIS | Details ... | 95 | 0.34 | 1.34 | 0.013 | 0.172 | 0.422 | 1216 | tags=25%, list=14%, signal=29% |
| 6 | HALLMARK\_UV\_RESPONSE\_DN | Details ... | 105 | 0.33 | 1.32 | 0.012 | 0.174 | 0.479 | 1817 | tags=30%, list=20%, signal=38% |
| 7 | HALLMARK\_COMPLEMENT | Details ... | 92 | 0.33 | 1.30 | 0.057 | 0.163 | 0.513 | 451 | tags=18%, list=5%, signal=19% |
| 8 | HALLMARK\_APICAL\_SURFACE | Details ... | 20 | 0.45 | 1.30 | 0.122 | 0.153 | 0.541 | 1409 | tags=35%, list=16%, signal=41% |
| 9 | HALLMARK\_HEDGEHOG\_SIGNALING | Details ... | 25 | 0.42 | 1.27 | 0.137 | 0.162 | 0.600 | 1634 | tags=36%, list=18%, signal=44% |
| 10 | HALLMARK\_IL6\_JAK\_STAT3\_SIGNALING | Details ... | 33 | 0.39 | 1.27 | 0.101 | 0.147 | 0.604 | 1571 | tags=30%, list=17%, signal=37% |
| 11 | HALLMARK\_IL2\_STAT5\_SIGNALING | Details ... | 105 | 0.31 | 1.23 | 0.106 | 0.176 | 0.702 | 1636 | tags=30%, list=18%, signal=37% |
| 12 | HALLMARK\_COAGULATION | Details ... | 53 | 0.32 | 1.17 | 0.175 | 0.259 | 0.861 | 1221 | tags=30%, list=14%, signal=35% |
| 13 | HALLMARK\_ESTROGEN\_RESPONSE\_EARLY | Details ... | 103 | 0.28 | 1.15 | 0.148 | 0.263 | 0.885 | 1604 | tags=24%, list=18%, signal=29% |
| 14 | HALLMARK\_WNT\_BETA\_CATENIN\_SIGNALING | Details ... | 27 | 0.37 | 1.14 | 0.248 | 0.262 | 0.897 | 1863 | tags=30%, list=21%, signal=37% |
| 15 | HALLMARK\_PROTEIN\_SECRETION | Details ... | 89 | 0.29 | 1.12 | 0.221 | 0.278 | 0.923 | 844 | tags=20%, list=9%, signal=22% |
| 16 | HALLMARK\_KRAS\_SIGNALING\_UP | Details ... | 84 | 0.28 | 1.12 | 0.200 | 0.268 | 0.928 | 1813 | tags=27%, list=20%, signal=34% |
| 17 | HALLMARK\_ANDROGEN\_RESPONSE | Details ... | 69 | 0.29 | 1.10 | 0.266 | 0.288 | 0.949 | 472 | tags=14%, list=5%, signal=15% |
| 18 | HALLMARK\_BILE\_ACID\_METABOLISM | Details ... | 54 | 0.23 | 0.83 | 0.851 | 0.899 | 1.000 | 2142 | tags=39%, list=24%, signal=51% |
| 19 | HALLMARK\_KRAS\_SIGNALING\_DN | Details ... | 32 | 0.22 | 0.72 | 0.914 | 0.961 | 1.000 | 2787 | tags=44%, list=31%, signal=63% |
Table: Gene sets enriched in phenotype **na **[plain text format]****
